# Kv1.1 channel subunits in the control of neurocardiac function

**DOI:** 10.1080/19336950.2019.1635864

**Published:** 2019-06-28

**Authors:** Edward Glasscock

**Affiliations:** Department of Biological Sciences, Southern Methodist University, Dallas, TX, USA

**Keywords:** Kv1.1, Kcna1, heart, action potential, epilepsy, SUDEP

## Abstract

Voltage-gated Kv1.1 potassium channel α-subunits are broadly expressed in the nervous system where they act as critical regulators of neuronal excitability. Mutations in the *KCNA1* gene, which encodes Kv1.1, are associated with the neurological diseases episodic ataxia and epilepsy. Studies in mouse models have shown that Kv1.1 is important for neural control of the heart and that *Kcna1* deletion leads to cardiac dysfunction that appears to be brain-driven. Traditionally, *KCNA1* was not believed to be expressed in the heart. However, recent studies have revealed that Kv1.1 subunits are not only present in cardiomyocytes, but that they also make an important heart-intrinsic functional contribution to outward K^+^ currents and action potential repolarization. This review recounts the winding history of discovery of *KCNA1* gene expression and neurocardiac function from fruit flies to mammals and from brain to heart and looks at some of the salient questions that remain to be answered regarding emerging cardiac roles of Kv1.1.

## Introduction

Voltage-gated potassium (Kv) channels are important regulators of membrane excitability in the brain and heart, controlling action potential initiation, propagation, shape, and repetitive firing properties in neurons and cardiomyocytes [1,2]. Many Kv channel proteins are expressed in both brain and heart tissues where they can exert a dual influence over neuronal and cardiac function and where channelopathies can potentially lead to a mixture of neurological and cardiac diseases [3]. One such neurocardiac Kv channel subunit is Kv1.1. Despite being the first mammalian Kv channel subunit to be cloned and linked to human disease, the expression and function of Kv1.1 in the heart has only recently come to light. In this review, the circuitous discovery of Kv1.1 in the heart is recounted while also discussing the neural control of cardiac function by Kv1.1. Research on Kv1.1 provides a model example of fruitful bench-to-bedside translational research, whereby basic science findings have repeatedly led to subsequent confirmatory findings in patients.

## Overview of Kv channel structure

Kv channels are composed of four α-subunit proteins that assemble as homo- or hetero-tetramers to form a membrane pore that allows the selective flux of K^+^ ions [4]. Each α-subunit protein is composed of six transmembrane segments (S1-S6), four of which (S1-S4) comprise a voltage sensor domain and two of which (S5-S6) make up the central pore region [5]. Linking S4 and S5 is a P-loop domain that confers ion selectivity [6]. In humans, the pore-forming Kv α-subunits are encoded by 40 different genes, which belong to 12 gene subfamilies (Kv1-Kv12), some of which exhibit alternative splicing and RNA editing [7]. The α-subunit tetramers form multiprotein complexes by co-assembling with up to four cytoplasmic auxiliary β-subunits, which influence channel gating and surface expression [8]. Thus, Kv channels represent a molecularly diverse group with an array of possible channel combinations.

## Discovery of Kv1.1 transcripts in brain

The first mammalian voltage-gated potassium channel α-subunit gene to be cloned, sequenced, and characterized was *Kcna1*, which originally was isolated from mouse brain and named MBK1 for mouse brain potassium channel 1 gene [9]. The successful initial cloning of *Kcna1* (MBK1) from mouse brain was soon followed several months later by the cloning and sequencing of highly homologous isoforms from rat cortical (RCK1) and hippocampal (RBK1) cDNA libraries, and then finally from human genomic DNA (HuKi or HK1) [10–12]. Because of the disparate and potentially confusing names given to these homologous channels identified in different species, a simplified nomenclature based on sequence relatedness was adopted that assigned a gene name of *Kcna1* (*KCNA1* in humans) and a channel protein name of Kv1.1 (K for potassium; v for voltage-dependent; and 1.1 for the first identified member of the channel subfamily 1) [13,14].

**Figure 1.**
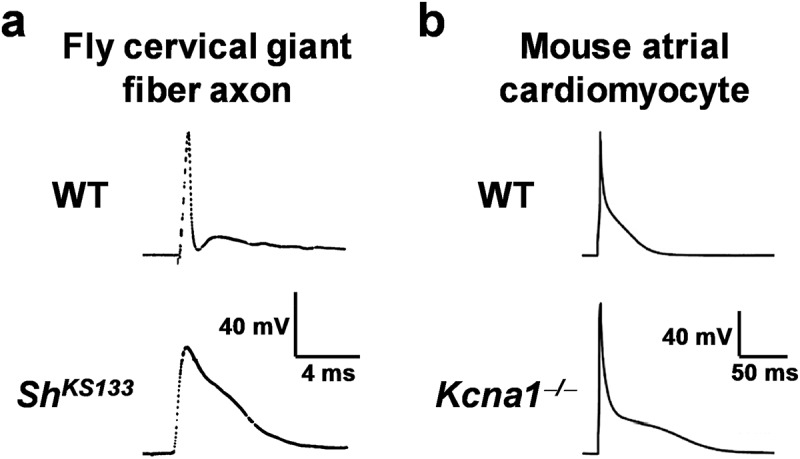
Action potential prolongation in *Shaker* flies and Kv1.1-deficient mice. (a) In mutant *Shaker* flies with the KS133 allele (*Sh^KS133^*), the cervical giant fiber axon exhibits prolonged action potentials compared to normal wildtype (WT) flies due to delayed repolarization (images reproduced with permission of Mark Tanouye [20]). (b) In *Kcna1*^–/ –^ mice lacking Kv1.1 channels, atrial cardiomyocytes exhibit significantly prolonged action potentials reminiscent of findings in the *Shaker* flies (images reproduced with permission [58]).

The breakthrough discovery of *Kcna1* was made possible by the prior positional cloning of the *Drosophila* ortholog of Kv1.1, the *Shaker* (*Sh*) gene [15–18], which provided the conserved nucleotide sequences needed to successfully screen mammalian cDNA libraries for similar transcripts [9]. The *Sh* locus was first identified and so named because *Sh* mutants exhibit leg-shaking behavior when exposed to ether anesthesia such as during fly sorting [19]. Prior to its cloning, *Sh* had been predicted to encode a potassium channel based on functional studies. In electrophysiology experiments, *Sh* mutants exhibited prolongation of action potentials in adult fly giant fiber axons (Figure 1(a)) and of neurotransmitter release at larval neuromuscular junctions, which could be mimicked in normal flies by addition of the potassium channel blocker 4-aminopyridine [20,21].

Despite their sequence similarities, the fly and mammalian orthologs of *Kcna1* exhibit some important differences at the levels of gene regulation and function. The *Drosophila Shaker* locus contains 21 exons that form a single primary transcript, which is alternatively spliced to generate several distinct K^+^ channel proteins with differing physiological properties [22,23]. In contrast, the mammalian *Kcna1* ortholog is intronless; therefore, mammals achieve Kv1 channel diversity by the presence of multiple gene loci [24]. Kv1 channel subunits can be broadly categorized into 2 functional groups: those that mediate non-inactivating delayed rectifier currents and those that mediate rapidly inactivating transient A-type currents. Shaker channels typically exhibit A-type currents due to the presence of a N-terminal protein domain that causes fast inactivation by a ball and chain mechanism [25,26]. Mammalian Kv1.1 channels lack this protein domain so they inactivate slowly unless complexed with another subunit that confers fast inactivation, such as a Kv1.4 α-subunit or a Kvβ1 subunit [27,28]. Therefore, the subunit stoichiometry of the channel complex determines the unique kinetics and gating properties of the channel. In the brain, Kv1.1 α-subunits form heterotetramers with Kv1.2, Kv1.4, and Kv1.6 [29]. The Kv1.6 α-subunits possess an N-type inactivation-prevention domain that opposes channel inactivation (i.e., the opposite of Kv1.4) to promote delayed rectifier currents [30]. Although they can form *in vitro*, Kv1.1 homotetramers have not been identified *in vivo*.

## Discovery of Kv1.1 mutations causing neurological disorders

**Figure 2.**
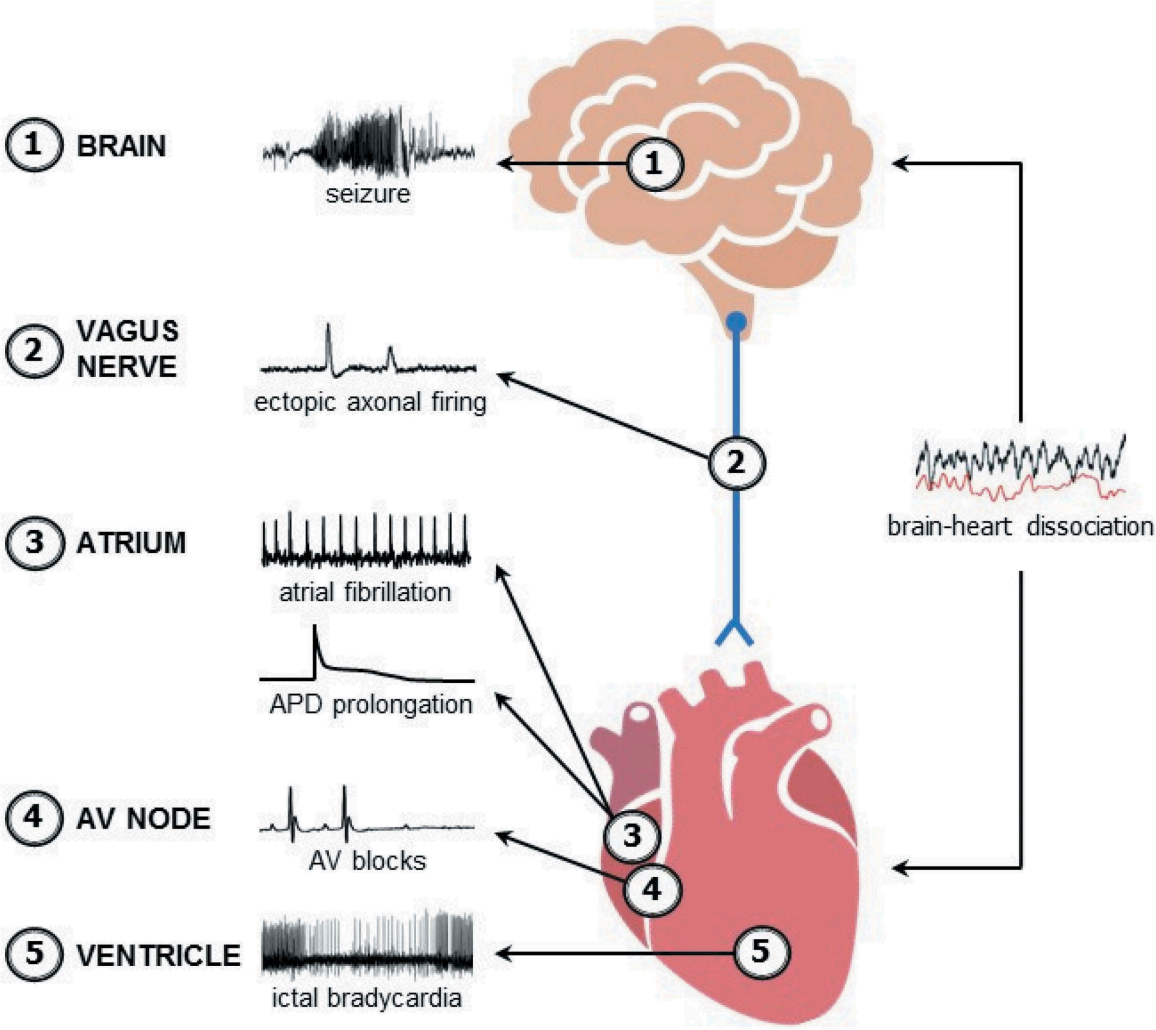
Summary of dysfunction along the brain-heart axis in *Kcna1*^–/–^ mice. *Kcna1*^–/–^ mice exhibit: (1) spontaneous tonic-clonic seizures which manifest as epileptiform electrographic activity in the brain [33]; (2) increased susceptibility to spontaneous ectopic action potential firing in the presence of 4-aminopyridine in myelinated vagus nerve axons [46]; (3) increased susceptibility to intracardiac-pacing induced atrial fibrillation and prolongation of action potential duration in atrial cardiomyocytes [57,58]; (4) increased frequency of atrioventricular (AV) nodal conduction blocks [35]; and (5) bradycardia during seizures [35,39]. In addition, analyses of brain-heart interaction dynamics reveals that *Kcna1*^–/–^ mice have significantly decreased brain-heart association, which could be indicative of abnormal uncoupling of brain and heart activity that increases risk of sudden death [47].

The first K^+^ channel gene directly linked to a human disease was *KCNA1*. Linkage studies and mutation analysis identified 4 different *KCNA1* dominant missense mutations across 4 families that caused the neurological disease episodic ataxia type 1 (EA1), which is characterized by periodic stress-induced incoordination and myokymia (i.e., muscle rippling) [31]. These findings were later verified and studied in a mouse model of episodic ataxia that was generated by engineering animals with the same V408A mutation identified in one of the patients [32]. Following the discovery of an association between *KCNA1* and EA1, *Kcna1* mutation was found to cause epilepsy, first in mice and then in humans. In mice, an engineered deletion of the entire *Kcna1* open reading frame to generate a null mutation (*Kcna1*^–/–^) resulted in a severe epilepsy phenotype, characterized by spontaneous tonic-clonic seizures that occurred several times daily and culminated in sudden death in about half of animals (Figure 2) [33]. The lethal seizures associated with the absence of Kv1.1 has led to *Kcna1*^–/–^ mice being widely used as a model for elucidating the mechanisms underlying sudden unexpected death in epilepsy (SUDEP) [34–36]. SUDEP is generally defined as the sudden unexpected death of someone with epilepsy, who is otherwise healthy, for which no obvious cause of death can be found [37]. SUDEP is the leading cause of epilepsy-related mortality, accounting for the second most years of potential life lost among all neurological disorders [38]. *Kcna1*^–/–^ mice recapitulate many of the risk factors and terminal neurocardiac patterns observed in human SUDEP victims including: (i) frequent seizures; (ii) generalized tonic–clonic seizures; (iii) early onset epilepsy; (iv) long duration of epilepsy; (v) young age; and (vi) seizure-associated cardiorespiratory arrest [33–36,39,40]. However, it should be noted that SUDEP is genetically complex and no single gene reaches genome-wide significance in exome sequencing studies [41,42].

The epilepsy phenotype associated with *Kcna1* mutations represents an illustrative example of successful bench-to-bedside scientific discovery. About a year after the initial discovery that *Kcna1* deletion causes epilepsy in mice, the first patients were identified with epilepsy due to *KCNA1* loss-of-function missense mutations. In addition to EA1, these patients exhibited complex partial seizures with secondary generalization [43]. More recently, patients without ataxia have also been identified that have tonic-clonic seizures due to *KCNA1* mutations [44]. Finally, a *de novo* copy number variant in *KCNA1* was identified in a SUDEP victim, suggesting that *KCNA1* mutations can increase susceptibility to SUDEP in humans [45].

## Neural control of cardiac function by Kv1.1

Studies into the mechanisms of SUDEP in *Kcna1*^–/–^ mice were the first to reveal cardiac abnormalities due to the absence of Kv1.1. In recordings of cardiac activity using electroencephalography (EEG) – electrocardiography (ECG) together or ECG telemetry alone, *Kcna1*^–/–^ mice exhibited seizure-evoked atrioventricular (AV) conduction blocks, bradycardia, and asystole (Figure 2) [35]. SUDEP events in *Kcna1*^–/–^ mice are associated with postictal bradycardia that progressively worsens leading to eventual cardiac arrest preceded by electrocortical silence [35,39]. EEG-ECG recordings also revealed that *Kcna1*^–/–^ mice have interictal cardiac abnormalities including an increase in the frequency of AV blocks (Figure 2) [35]. These AV blocks can be eliminated by either autonomic blockade with co-administration of atropine and propranolol or selective parasympathetic blockade with atropine alone, but not by administration of the sympathetic blocker propranolol by itself [35]. Thus, the cardiac conduction blocks in *Kcna1*^–/–^ mice appear to be brain-driven, specifically by the parasympathetic branch of the autonomic nervous system.

Measurements of heart rate variability (HRV) and vagus nerve function have revealed additional evidence of altered parasympathetic signaling underlying the cardiac abnormalities in *Kcna1*^–/–^ mice. Kv1.1 channels are expressed in the myelinated axons of the vagus nerve [35,46]. When Kv1.1 is absent, myelinated vagal axons exhibit increased susceptibility to spontaneous ectopic firing in the presence of the Kv channel blocker 4-aminopyridine, indicative of hyperexcitability (Figure 2) [46]. Although *in vivo* heart rates (HR) appear mostly unchanged in *Kcna1*^–/–^ mice, they do exhibit increases in the root mean square of successive beat-to-beat differences, a heart rate variability (HRV) measure of parasympathetic tone, suggesting increased vagal influence [35,47,48]. Unilateral transection of the vagus nerve in *Kcna1*^–/–^ mice moderately increases lifespan by about 1–2 weeks, further implicating vagal mechanisms in SUDEP in this model [39].

In addition to vagal-specific aspects of cardiac control, *Kcna1*^–/–^ mice also exhibit altered brain-heart interaction dynamics in EEG-ECG recordings. Typically, EEG and ECG data are analyzed separately when performing concurrent EEG-ECG recordings. However, a novel type of mathematical analysis, termed interaction dynamics, can be applied to the data to evaluate the degree of association between the brain (EEG) and heart (ECG) biosignals to reveal signs of neurocardiac dysfunction [47]. Normally, wild-type (WT) mice exhibit a negative correlation between the duration of the cardiac RR intervals of the ECG and the signal complexity (i.e., entropy) of the EEG, implying that HR and the entropy of brain signals tend to increase and decrease together [47]. In *Kcna1*^–/–^ mice, this pattern is mostly abolished and the degree of association between EEG and ECG signals is significantly reduced, even during periods without seizures (Figure 2) [47]. Although the specific pathophysiology underlying this brain-heart dissociation needs to be further elucidated, the data points to a potential uncoupling of brain and heart activity in *Kcna1*^–/–^ mice, which could be indicative of deleterious dysregulation of neural control of the heart and could increase risk for sudden death.

## Discovery and re-discovery of Kv1.1 in the heart

Numerous older studies described *Kcna1* expression in the heart, but these reports were largely dismissed at the time leading to the widespread assertion that Kv1.1 is absent in heart. The first report of Kv1.1 in the heart was in the year 1991 when Roberds and Tamkun used Northern blotting to detect *Kcna1* mRNA in rat atrial tissue [49]. However, the authors hypothesized that the transcripts might arise from neuronal cells since the abundance was so low [49]. About 3 years later, Dixon and McKinnon attempted unsuccessfully to detect *Kcna1* mRNA in rat atria and ventricles using an RNase protection assay method, which led them to conclude that the previous positive detection of *Kcna1* mRNA in the atria by Roberds and Tamkun was likely due to contamination by neural tissues [50]. Following up on this work in rat heart, Brahmajothi *et al* used *in situ* hybridization to detect *Kcna1* mRNA in cardiomyocytes isolated from ferret hearts, including sinoatrial nodal, atrial, and ventricular cells [51]. They rationalized that their fluorescent *in situ* hybridization technique permitted detection of low abundance *Kcna1* transcripts due to the increased sensitivity of the method compared to RNase protection in the Dixon and McKinnon study. However, they also allowed for the possibility of species differences in channel expression that could explain the difference in results [51]. Finally, in 2001, London *et al* used Northern blots to detect *Kcna1* mRNA in whole mouse heart, but they attributed this to non-myocytes since the expression bands were faint [52]. Because of the inconsistent results in these early studies and the assumption that positive detection was due to neuronal contamination, over time the accepted dogma became that *Kcna1* was not expressed or functional in cardiomyocytes.

With the advent of highly sensitive PCR-based mRNA detection methods, such as real-time reverse transcriptase PCR (RT-PCR), multiple studies began to report cardiac *Kcna1* transcripts using these new techniques, which began to cast doubt on the earlier conclusions that Kv1.1 is absent in the heart. Using real-time RT-PCR, Marionneau *et al* reported *Kcna1* expression in the atria, ventricles, sinoatrial node (SAN), and atrioventricular node of mouse heart corroborating the previous work done in ferrets [51,53]. In a study of channel transcripts that are remodeled during chronic heart rate reduction, real-time RT-PCR revealed that *Kcna1* mRNA levels are not only detectable in the SAN, but that they increase more than any other voltage-gated K^+^ channel in response to bradycardia [54]. In a comprehensive analysis of ion channel expression using a large-scale real-time quantitative RT-PCR assay, Harrell *et al* found *Kcna1* to be the most abundantly expressed member of the Kv channel subfamily in mouse ventricles throughout perinatal development [55]. However, despite these successes using RT-PCR based methods in mouse heart, a study utilizing high-throughput real-time RT-PCR in human heart samples still found that Kv1.1 expression frequently fell below the threshold for detection [56]. Although newer and more sensitive PCR-based methods facilitated the reliable detection of *Kcna1* mRNA in mouse hearts, Kv1.1 protein expression and function in the heart remained to be demonstrated.

In a series of papers by Glasscock *et al*, Kv1.1 protein expression was demonstrated in the heart for the first time [35,57]. Using western blotting, Kv1.1 protein was detected in whole mouse heart; however, the expression band was relatively weak and only visible in lysates with >100 μg of total protein [35]. In a follow up study, immunocytochemistry was used to reveal Kv1.1 localization in isolated atrial and ventricular myocytes from mouse hearts [57]. In atrial cells, Kv1.1 immunoreactivity showed a predominantly punctate intracellular staining pattern, whereas in ventricular cells, the staining pattern was of weaker intensity with clustering consistent with transverse (T) tubules [57]. The greater intensity of Kv1.1 immunoreactivity in atrial cells correlates with quantitative RT-PCR experiments which show that *Kcna1* transcripts are about 10-fold more abundant in atrial myocytes compared to ventricular myocytes [57].

The demonstration of Kv1.1 expression at the protein level in mouse heart spurred additional investigation into the presence of Kv1.1 in human heart. First using RT-PCR and then immunoblotting, Kv1.1 mRNA and protein were detected in human atrial appendages [57]. Immunocytochemistry experiments using isolated human atrial myocytes revealed Kv1.1-positive immunoreactivity in a mostly intracellular staining pattern similar to results in mice, but also with some striations consistent with Kv1.1 localization at Z-lines where the T tubules reside [57]. Just as Kv1.1-associated epilepsy was discovered first in mice and then in humans, cardiac Kv1.1 expression was first demonstrated in mice, which led to its subsequent discovery in human heart, providing another example of successful bench-to-bedside discovery.

## Intrinsic control of cardiac function by Kv1.1

The discovery of Kv1.1 in the heart opened the door to the possibility that Kv1.1 subunits make a direct intrinsic contribution to cardiac function, not only indirectly via the autonomic nervous system. The first indication that Kv1.1 could be important for intrinsic cardiac function came from experiments in *Kcna1*^–/–^ mice using programmed electrical stimulation of the heart [57]. When the hearts of *Kcna1*^–/–^ mice were stimulated using intracardiac burst pacing, they exhibited increased susceptibility to atrial fibrillation (AF; Figure 2). However, it should be noted that these experiments were performed *in vivo* with an intact nervous system, so neural influences on arrhythmogenesis cannot be entirely ruled out. To test whether Kv1.1 channels contribute to cardiac currents, patch-clamp electrophysiological recordings were performed in isolated human atrial myocytes to measure outward K^+^ currents [57]. In the presence of dendrotoxin-K (DTX-K), a specific blocker of Kv1.1 subunits, the peak and late outward K^+^ currents were significantly reduced, providing the first demonstration of functional expression of Kv1.1 in the heart and suggesting the subunits contribute to cardiac repolarization. When Kv1.1 protein was originally detected in human heart, the levels were found to significantly increase by about 75% in patients with chronic AF (cAF), suggesting disease-associated alterations in expression [57]. When outward K^+^ currents were measured in human atrial myocytes isolated from cAF patients, the DTX-K-sensitive portion of the current was increased two- to three-fold compared to patients in sinus rhythm [57]. This augmentation in current correlated with the increase in protein levels in cAF patients, suggesting that Kv1.1 undergoes electrical remodeling that may contribute to cAF pathomechanisms.

In a recent study, Si *et al* performed patch-clamp recordings of atrial cardiomyocytes isolated from *Kcna1*^–/–^ mice revealing that Kv1.1-mediated currents regulate atrial repolarization and action potential morphology [58]. As observed in human atrial cells, WT mouse atrial myocytes also exhibited a significant DTX-K-sensitive outward K^+^ current component indicative of a contribution by Kv1.1-containing channels [58]. Importantly, DTX-K-sensitive outward K^+^ currents were absent in *Kcna1*^–/–^ cells which lack Kv1.1, demonstrating the specificity of DTX-K for Kv1.1 and suggesting that the DTX-K-sensitive currents recorded in human cells were indeed indicative of functional Kv1.1 subunits and not some off target effect [58]. In addition to measuring outward K^+^ currents, Si *et al* also recorded the effects of Kv1.1-deficiency on cardiac action potential morphology for the first time. *Kcna1*^–/–^ atrial myocytes exhibited significantly prolonged action potential durations (APD) at 90% repolarization (APD_90_) compared to WT cells (Figures 1(b) and 2). Interestingly, the already prolonged APD durations in *Kcna1*^–/–^ mice were significantly longer in cells from the right atrium versus the left atrium, suggesting Kv1.1 may contribute to interatrial differences in repolarization that are present in mammals to maintain normal right-to-left atrial electrical conduction [58–60]. APD prolongation could also be induced in WT cells by administration of DTX-K, but not in *Kcna1*^–/–^ cells, which further demonstrates the specificity of the toxin for Kv1.1 [58]. These electrophysiology studies showed that not only is the *Kcna1* gene expressed in cardiomyocytes, but that channels containing Kv1.1 are functional in the heart and critical for cardiac repolarization and action potential waveform properties which could influence arrhythmogenesis. Thus, Kv1.1 represents a novel cardiac K^+^ channel.

## Unanswered questions about Kv1.1 in the heart

Elucidation of the role of Kv1.1 in the heart is still in its early stages and several important questions remain to be explored to fully understand the effects of Kv1.1 dysfunction on cardiac disease and arrhythmia susceptibility. Mammalian hearts express several prominent repolarizing K^+^ currents mediated by voltage-gated K^+^ channels, including transient outward (*I_to_*) and delayed rectifier currents (*I_K_*) [61]. The primary K^+^ channel subunits that encode these various cardiac repolarizing currents are generally thought to be known and have been extensively characterized. Maybe Kv1.1 contributes to one of these already identified currents or perhaps it encodes a subtle current component not yet described. Another important aspect of the function of Kv1.1-containing channels is subunit stoichiometry. Do Kv1.1 channels form hetero-tetramers with one of the previously identified cardiac channels to mediate one of these well known cardiac currents? If Kv1.1 forms functional homo-tetrameric channels in the heart, it would be the first *in vivo* demonstration of Kv1.1 homo-tetramers in any cell or tissue type. The identity of interacting auxiliary β-subunits will also need to be determined, especially since these can impact channel gating and trafficking.

Although *Kcna1* deletion in mice has been associated with increased AF susceptibility, human *KCNA1* mutations causing AF have not yet been reported. The lack of human patients with AF due to *KCNA1* variants could be due to sampling bias or it could be indicative of a species difference in the arrhythmogenic roles of Kv1.1 in the heart. Given the bench-to-bedside trajectory that Kv1.1 studies have taken, it would be appropriate if AF-causing *KCNA1* mutations were identified following the original discovery of AF susceptibility in the *Kcna1*^–/–^ mouse model. In addition, the actual mechanism whereby Kv1.1 deficiency or dysfunction can increase atrial arrhythmia susceptibility requires elucidation. One possibility is that atrial APD prolongation related to Kv1.1 mutation promotes early afterdepolarizations that increase the likelihood of AF. To date, studies of Kv1.1 function and expression in the heart have focused on the atria. However, Kv1.1 mRNA and protein have been detected in the ventricles using RT-PCR and immunocytochemistry, respectively [35,51,53,55,57]. Kv1.1 channels would be expected to contribute to repolarization in ventricular cells as observed in atria, but possibly to a lesser extent since mouse ventricular expression levels are ~10% of the levels in atria. Since the ventricles can be a source of lethal arrhythmias and *Kcna1*^–/–^ mice exhibit seizure-related sudden death, determining the effects of Kv1.1 mutations on ventricular arrhythmia susceptibility is especially important. Future studies will also need to address the expression of Kv1.1 in human ventricles, which may be substantially less than atria as suggested by expression studies which report low abundance mRNA levels [56].

Finally, the relationship between cardiac Kv1.1 channels and SUDEP deserves additional investigation. Although the discovery of functional Kv1.1 channels in the heart raises the possibility of an intrinsic cardiac contribution to SUDEP in the *Kcna1*^–/–^ mouse model, it does not necessarily rule out brain-driven mechanisms as the primary cause. One possibility is that the absence of Kv1.1 in the heart may not be required for SUDEP, but that it renders the heart a vulnerable substrate for lethal cardiac arrhythmias triggered by seizures. Thus, the relationship between Kv1.1-deficient brain and heart needs to be examined further to determine the relative contributions of the two tissues to SUDEP and their potentially deleterious synergistic interactions.

## Conclusions

In the span of the last 30 years, Kv1.1 channels have been discovered in the heart, dismissed as absent in the heart, and then re-discovered in the heart. Although the neural roles of Kv1.1 are well known, potential cardiac roles of Kv1.1 are just now being elucidated. Studies of the *Kcna1* gene have been a model example of fruitful bench-to-bedside discovery. *Kcna1* mutations were first found to cause epilepsy in mice which led to their discovery in human epilepsy patients. *Kcna1* mutations were first found to increase susceptibility to SUDEP in mice, which then led to the successful identification of a SUDEP patient with a *KCNA1* variant that was a principal risk factor in their death. *Kcna1* expression was first found in rodent hearts which led to its subsequent discovery in human heart. Given this bench-to-bedside pattern of discovery, future studies in the lab on the neurocardiac roles of Kv1.1 are almost certain to uncover additional unexpected insights that will continue to get translated into potentially life-saving tools in the clinic.
